# Tubular Cell Dropout in Preimplantation Deceased Donor Biopsies as a Predictor of Delayed Graft Function

**DOI:** 10.1097/TXD.0000000000001168

**Published:** 2021-06-18

**Authors:** Zachary M. Avigan, Nikhil Singh, Judith A. Kliegel, Marlene Weiss, Gilbert W. Moeckel, Lloyd G. Cantley

**Affiliations:** 1 Section of Nephrology, Department of Internal Medicine, Yale University School of Medicine, New Haven, CT.; 2 Department of Pathology, Yale University School of Medicine, New Haven, CT.

## Abstract

**Background.:**

Delayed graft function (DGF) affects over 25% of deceased donor kidney transplants (DDKTs) and is associated with increased cost, worsened graft outcomes, and mortality. While approaches to preventing DGF have focused on minimizing cold ischemia, donor factors such as acute tubular injury can influence risk. There are currently no pharmacologic therapies to modify DGF risk or promote repair, in part due to our incomplete understanding of the biology of preimplantation tubular injury.

**Methods.:**

We collected intraoperative, preimplantation kidney biopsies from 11 high-risk deceased donors and 10 living donors and followed transplant recipients for graft function. We performed quantitative high-dimensional histopathologic analysis using imaging mass cytometry to determine the cellular signatures that distinguished deceased and living donor biopsies as well as deceased donor biopsies which either did or did not progress to DGF.

**Results.:**

We noted decreased tubular cells (*P* < 0.0001) and increased macrophage infiltration (*P* = 0.0037) in high-risk DDKT compared with living donor biopsies. For those high-risk DDKTs that developed postimplant DGF (n = 6), quantitative imaging mass cytometry analysis showed a trend toward reduced tubular cells (*P* = 0.02) and increased stromal cells (*P* = 0.04) versus those that did not (n = 5). Notably, these differences were not identified by conventional histopathologic evaluation.

**Conclusions.:**

The current study identifies donor tubular cell loss as a precursor of DGF pathogenesis and highlights an area for further investigation and potential therapeutic intervention.

## Abstract 2

Supplemental Digital Content is available in the text.

## INTRODUCTION

Delayed graft function (DGF) is most commonly defined as dialysis requirement within 1 week of transplant.^1-4^ DGF incidence in the United States has risen to as high as 27.5% in 2018,^1,5^ likely due to increasing demand and more aggressive transplantation of extended criteria and high-risk organs.^6,7^ Various studies have shown a deleterious effect of DGF on outcomes, including rejection, graft survival, mortality, length of hospital stay, and cost.^6,8-12^ Efforts to prevent DGF have centered on minimizing cold ischemia time and optimizing organ preservation, which represent important modifiable risk factors.^1,13,14^ However, donor factors such as donation after circulatory death (DCD), age, and terminal creatinine also play a significant role.^13,15-18^ We hypothesize that molecular characterization of donor tissue will advance our mechanistic understanding of DGF and may lead to the development of therapies to improve management and prevent lasting damage to the graft.

DGF is due primarily to ischemia-reperfusion injury (IRI),^15^ and morphologic features of ischemic tubular injury have been identified in protocol biopsies of DGF kidneys as late as 6 months posttransplantation.^19^ Unfortunately, histologic evaluation alone of preimplant graft biopsies does not consistently correlate with posttransplant function and outcomes.^20^ Further work is needed to better elucidate mechanisms of cellular injury contributing to peritransplant IRI, but this analysis has been limited by the availability of biopsy tissue and the throughput of conventional analysis techniques. To increase the depth of information gained from a single biopsy, several groups have applied single-cell transcriptional sequencing methods to study ischemic and inflammatory transplant injury.^21,22^ However, single-cell transcriptomic techniques exclude spatial information and therefore cannot define regional heterogeneity or individual cellular interactions. Additionally, protease dissociation steps may preferentially release particular cell compartments and thus bias data toward certain cell types.^23,24^ Therefore, quantitative cellular analysis of intact biopsy sections could more accurately measure changes in individual cell subsets.

Imaging mass cytometry (IMC) is a highly multiplexed tissue imaging technology that allows for simultaneous detection of up to 42 protein markers on an individual formalin-fixed, paraffin-embedded biopsy section.^25,26^ The tissue is labeled with a cocktail of antibodies specific for antigens of interest, each conjugated to a unique heavy-metal isotope, followed by sequential pulsed laser ablation of 1-μm^2^ regions. Each resulting aerosolized plume undergoes time-of-flight mass spectrometry analysis to determine the presence and abundance of each antibody, and data are reconstructed to form a quantitative map of each protein with 1-μm resolution. This approach vastly increases the dimensionality and throughput of protein expression data compared with serial immunofluorescence while preserving spatial information.^27^ Our group has previously developed and validated an antibody panel defining the cellular diversity of reference human kidney tissue and an automated kidney-specific data analysis pipeline.^28^

In this study, we extended our validated IMC pipeline to investigate cellular changes preceding DGF. We obtained preimplantation biopsies from high-risk deceased donor kidney transplants (DDKTs) and used preimplantation biopsies from living donor kidney transplants (LDKTs) as healthy controls. Biopsies were interrogated for signs of injury and differences in tubular, stromal, endothelial, and immune populations between living and deceased donors and between those that subsequently developed immediate graft function (IGF) versus DGF. These data represent the first use of IMC to study human kidney disease and provide a better understanding of the cellular basis for a predisposition of transplanted kidneys to developing DGF.

## MATERIALS AND METHODS

### Patient Recruitment and Tissue Collection

Kidney transplant recipients were recruited from June 2018 through May 2019 under a protocol approved by the Yale University institutional review board (Protocol 2000022915). Informed consent was obtained for all patients before enrollment. For each participant, an 18-gauge core or wedge biopsy was collected intraoperatively during organ preparation and processed as previously described.^28^

Eighteen LDKT recipients and their corresponding donors were enrolled, with biopsies collected after approximately 5 minutes of cold ischemia. Fifteen samples were sectioned, with 3 biopsies excluded after processing due to inadequate size.

Adult DDKT recipients were identified for DGF risk >25% based on recipient and donor data using the calculator developed by Irish et al^16^ (Figure S1, SDC, http://links.lww.com/TXD/A333). A DGF risk cutoff of 25% was selected as it was associated with significantly decreased graft survival in that study.^16^ Forty-four DDKT recipients were assessed for eligibility, and 23 were excluded due to low DGF risk (20/44), simultaneous liver or dual kidney transplant (2/44), or duplicate donor with another patient (1/44). Of 21 eligible recipients, 16 consented to participate, of whom 7 developed DGF defined by dialysis requirement within 1 week of transplantation (7/16, 43.8%), similar to the group’s calculated DGF risk of 39.0% ± 10.6%.^16^

Based on our prior experience that ~10% of IMC regions would not yield analyzable data, 11 LDKT and 13 DDKT biopsies were selected at random for cortical ablation and labeling, with a goal of 10 per group. After hybridization, 3 samples (1 LDKT, 2 DDKT) were excluded due to large areas with unrecorded or nonspecific data. Ten LDKT biopsies were analyzed, with donor data shown (Table 1). Living donors all had estimated glomerular filtration rate >60 mL/min, and no LDKT recipients developed DGF, consistent with low rates nationally.^5^ The total analyzed tissue area was 10.26 mm^2^. Eleven DDKT samples (6/11 with subsequent DGF) were analyzed, with clinical data shown for both donors (Table 2) and recipients (Table 3). The total analyzed tissue area was 14.74 mm^2^ (DGF: 6.98 mm^2^).

**TABLE 1.** LDKT donor clinical data

Age	Male	HTN	Diabetes	Serum Cr (mg/dL)	eGFR (mL/min)
29	N	N	N	0.76	>60
37	Y	N	N	1.05	>60
64	N	N	N	0.78	>60
62	N	N	N	1.00	>60
28	N	N	N	0.69	>60
57	N	Y	N	0.70	>60
38	N	N	N	0.57	>60
33	N	N	N	0.58	>60
62	Y	Y	N	0.75	>60
43	N	N	N	0.81	>60
45.3 (14.5)	2/10	2/10	0/10	0.77 (0.16)	10/10

Summary data shown as mean (SD) where applicable.

Cr, creatinine; eGFR, estimated glomerular filtration rate; HTN, hypertension; LDKT, living donor kidney transplant..

**TABLE 2.** DDKT donor and transplant data

	Age	Male	DCD	HTN	Diabetes	Terminal Cr (mg/dL)	KDPI	Pump	WIT (h)	CIT (h)
**IGF**	61	N	N	N	N	0.90	80%	Y	-	17:34
	52	Y	N	N	Y	0.60	80%	Y	-	9:01
	73	Y	N	Y	Y	1.00	100%	N	-	23:03
	41	Y	Y	Y	N	0.72	48%	Y	0:12	6:36
	29	Y	N	N	N	3.19	22%	Y	-	25:48
	51.2 (17.1)	4/5	1/5	2/5	2/5	1.28 (1.08)	66.0 (30.9)%	4/5	-	16:24 (8:26)
**DGF**	22	N	N	N	N	0.70	8%	N	-	14:56
	24	Y	Y	N	N	0.80	17%	Y	0:10	15:19
	54	Y	Y	Y	N	0.53	78%	Y	0:18	21:09
	61	Y	Y	N	N	0.50	79%	Y	0:12	19:35
	49	Y	Y	Y	N	1.20	62%	N	0:16	23:30
	32	Y	N	N	N	1.11	52%	N	-	17:20
	40.3 (16.5)	5/6	4/6	2/6	0/5	0.81 (1.08)	49.3 (30.4)%	3/6	-	18:38 (3:23)
***P***	0.32	1.0	0.24	1.0	0.18	0.39	0.39	0.55	-	0.60

Summary data shown as mean (SD) where applicable, with *P* comparing IGF and DGF groups shown.

CIT, cold ischemia time; Cr, creatinine; DCD, donation after circulatory death; DGF, delayed graft function; HTN, hypertension; IGF, immediate graft function; KDPI, kidney donor profile index; WIT, warm ischemia time.

**TABLE 3.** DDKT recipient clinical data

	Age	Male	PRA	BMI	HLA mismatches	DGF risk
**IGF**	66	N	93%	35.0	5/6	40%
	36	N	94%	27.0	6/6	29%
	66	Y	0%	23.9	5/6	39%
	25	N	90%	20.6	4/6	34%
	64	N	59%	32.8	5/6	53%
	51.4 (19.5)	1/5	67.2 (40.3)%	27.9 (6.0)	5/6	39.0 (9.0)%
**DGF**	41	Y	0%	35.0	5/6	26%
	26	Y	0%	29.1	5/6	40%
	47	Y	0%	18.0	5/6	52%
	70	Y	0%	23.3	6/6	61%
	59	Y	0%	21.5	6/6	53%
	45	Y	0%	32.4	6/6	27%
	48.0 (15.2)	6/6	0 (0)%	26.6 (6.7)	5.5/6	43.2 (14.6)%
***P***	0.76	**0.02**	**0.003**	0.74	0.38	0.58

Summary data shown as mean (SD) where applicable, with *P* comparing IGF and DGF groups shown for each variable.

BMI, body mass index; DDKT, deceased donor kidney transplant; DGF, delayed graft function; IGF, immediate graft function; PRA, panel reactive antibody.

### Pathology

A renal pathologist (GWM) blinded to tissue identity reviewed 1 H&E and 1 Trichrome stained section from each biopsy, with histopathologic scoring shown (Table 4). Each biopsy was scored at ×100 magnification using a raster grid consisting of a matrix of 10 rows and 10 columns of equal-sized squares (100 total squares per grid). Squares overlying renal cortex were counted for individual features of globally sclerosed glomeruli, interstitial fibrosis and tubular atrophy, interstitial infiltrate, and intimal thickening. Tubular injury was scored in each square by the presence of 3 features: loss of brush border, tubular dilation, and epithelial flattening. Squares containing each type of injury (eg, interstitial fibrosis and tubular atrophy, tubular injury) were divided by the total number of squares reviewed and converted into a scoring scheme as follows: none (<5% of area involved), mild (6%–25%), moderate (26%–50%), and severe (>50%). Arteriolar hyalinosis was scored as mild when at least 1 arteriole was involved, moderate when >1 arteriole was involved, and severe when multiple arterioles had circumferential involvement.^29^

**TABLE 4.** Histopathologic scoring

	Grade	ATI	IFTA	Infiltrate	Glom. sclerosis	Intim. sclerosis	Hyalinosis
**LDKT**	Mild	**6/10**	5/10	1/10	5/10	2/10	0/10
	Moderate	**2/10**	0/10	0/10	1/10	1/10	0/10
**DDKT**	Mild	**4/11**	4/11	1/11	4/11	2/11	1/11
	Moderate	**7/11**	1/11	0/11	0/11	1/11	1/11
***P* Value**		**0.08**	0.56	0.94	0.39	0.99	0.37
**IGF**	Mild	1/5	2/5	1/5	3/5	1/5	1/5
	Moderate	4/5	1/5	0/5	0/5	1/5	1/5
**DGF**	Mild	3/6	2/6	0/6	1/6	1/6	0/6
	Moderate	3/6	0/6	0/6	0/6	0/6	0/6
***P***		0.30	0.45	0.25	0.14	0.49	0.23

Histopathologic scoring of LDKT vs DDKT biopsies and of IGF vs DGF biopsies by the Banff criteria for preimplantation biopsies.^29^

The number of biopsies from each group receiving a score of mild or moderate is shown for each category (no biopsy received a grade of severe in any category) with associated *P*. Note that for tubular injury, the majority of LDKT biopsies had a mild grade while DDKT biopsies predominantly showed moderate injury; however, there was no appreciable difference in tubular injury scoring between IGF and DGF samples.

ATI, acute tubular injury; DDKT, deceased donor kidney transplant; DGF, delayed graft function; Glom, glomerular; IFTA, interstitial fibrosis and tubular atrophy; IGF, immediate graft function; Intim, intimal; LDKT, living donor kidney transplant.

### IMC Analysis and Validation

Antibody validation and IMC immunolabeling and data analysis were performed as previously described.^28^ In brief, one 5-μm thick section from each biopsy within 1–3 sections of the scored H&E and Trichrome slides was labeled using a cocktail of metal-conjugated antibodies (Table S1, SDC, http://links.lww.com/TXD/A333). Biopsies were then ablated and imaged via the Nd:YAG 213 nm laser in the Hyperion Imaging System (Fluidigm Corporation) with 1-μm pixel size, with each aerosolized plume analyzed in a mass cytometer. Pixel classification was performed on resulting antibody images in ilastik (version 1.3.2)^30,31^ to define pixels as belonging to nuclei; membranes of tubular, endothelial, or interstitial cells; or background. Cell segmentation was performed in CellProfiler (version 3.1.8)^32^ using size-gated nuclei to define a cell and the surrounding membrane pixels to define cell borders. Finally, cell phenotyping was performed in histoCAT (version 1.76),^33^ an open-source program designed for analysis of mass cytometry data, using the Phenograph algorithm,^34^ with each cell cluster manually annotated for phenotypic identity by canonical protein expression.

For each biopsy, multiple regions were selected to span the depth of renal cortex beginning 400 μm from the renal capsule with a width of 400–600 μm and cortico-medullary depth of ~1000 μm per region. Slides were labeled on consecutive days in batches of 6–8 samples and then imaged over the course of approximately 1 month, with sample order randomized.

Validation of the analysis pipeline on injured tissue by manual cell counting was performed in representative regions from 4 deceased donor samples (3/4 DGF) as previously described.^28^

### Statistics

For patient data, continuous variables were compared by the Welch’s t-test, binary variables by the Fisher’s exact test, and HLA mismatches by the chi-square test. Histopathologic scoring was compared using the chi-square test. For comparison of the 4 repeated LDKT samples to the reference data set and for validation comparing manually counted and calculated cell populations, the Wilcoxon matched-pairs signed-rank test was used. Quantitative IMC data were compared by the Welch’s t-test. Twelve cell populations were compared across samples, and a Bonferroni-corrected significance cutoff of 0.004 was therefore used, with additional trends shown using a cutoff of 0.05. Reported values represent means with standard deviations.

## RESULTS

### High-risk Deceased Donors Exhibit Tubular Cell Loss With Increased Macrophages

**FIGURE 1.**
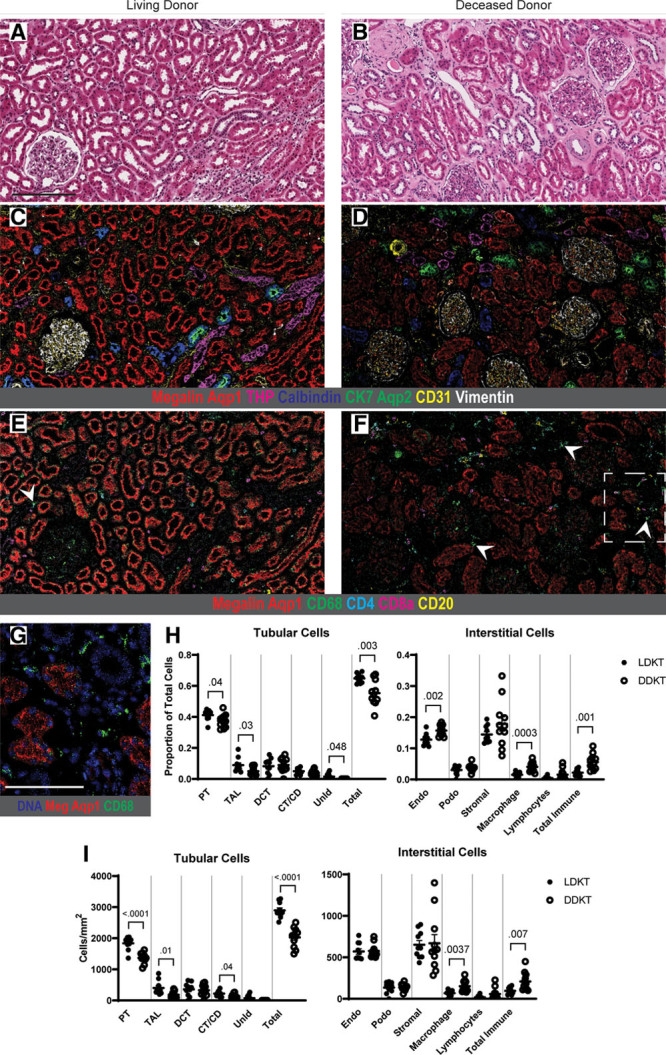
IMC analysis demonstrates quantitative and qualitative differences between LDKT and high-risk DDKT. (A, B) Representative H&E images from sections of living donor (A) and deceased donor (B) tissue. (C, D) Adjacent sections labeled by IMC for samples shown in (A, B), pseudocolored with a selection of antibody channels highlighting tubular and glomerular structures. The DDKT image has reduced tubular area and labeling intensity, particularly in the megalin-positive, aquaporin-1-positive proximal tubule. (E, F) Identical IMC regions, pseudocolored with antibodies highlighting the immune cell infiltrate. Note the increased immune cell abundance in the deceased donor sample (F), particularly CD68-positive macrophages, with a few example cells identified by arrowheads. (G) Boxed inset from (F) showing nuclei and CD68 to identify individual macrophages in the DDKT biopsy. (H) Cellular proportions of tubular and interstitial populations in LDKT and DDKT samples. (I) Cells per mm^2^ of analyzed tissue area for tubular and interstitial populations showing significantly reduced tubular cells, specifically in proximal tubule, as well as increased macrophage infiltration in high-risk deceased donor samples compared with living donor biopsies. All *P* values <0.05 shown, and bars show means ± standard errors of the mean. Scale bars: 200 μm (A), 100 μm (G). A gamma value of 1.0 was used for all protein channels in IMC images. Aqp1, aquaporin-1; Aqp2, aquaporin-2; CK7, cytokeratin-7; CT/CD, connecting tubule/collecting duct; DCT, distal convoluted tubule; DNA, DNA intercalator; Endo, endothelium; Meg, megalin; Podo, podocytes; PT, proximal tubule; TAL, thick ascending limb of loop of Henle; THP, Tamm-Horsfall protein; UnID, unidentified tubular cells.

Most LDKT biopsies had mild histopathologic changes (Table 4, Figure 1A) and qualitatively recapitulated the findings of our previous IMC-based reference kidney analysis, with ready detection of tubular, glomerular, and vascular structures (Figure 1C) and only rare detection of immune cells (Figure 1E). Quantitative comparison of the current LDKT samples against our previously published reference cohort^28^ showed no significant differences in any cell phenotype (Figure S2A and B, SDC, http://links.lww.com/TXD/A333) as well as no differences in paired comparisons of the 4 biopsies included in both studies (Figure S2C, SDC, http://links.lww.com/TXD/A333).

Before analysis of DDKT samples, we validated the use of our data analysis methodology on injured tissue. Four DDKT biopsies were subjected to IMC imaging followed by manual identification of over 8,000 cells in representative regions using 8 protein markers defining cell phenotype. This was compared with cell identification and quantitation using the automated pipeline. Consistent with our previous experience,^28^ automated quantitation successfully identified 96.0% ± 1.8% of overall cell number as defined by manual counting, with no significant difference in the proportion of any cell phenotype (Figure S2D, SDC, http://links.lww.com/TXD/A333). In particular, 99.5% ± 4.6% of tubular cells were correctly identified by our automated pipeline when compared with manual adjudication, suggesting that differences in protein expression between reference and injured tissue did not impair detection or classification of renal cell populations.

As compared with LDKT kidneys, DDKT samples showed a trend toward more significant tubular injury by histopathologic scoring, with 7/11 graded as moderate, although this did not reach statistical significance (*P* = 0.08) (Table 4, Figure 1B). By IMC, deceased donor biopsies had reduced cellular area and labeling intensity across tubular populations, most notably in the proximal tubule (PT; megalin-positive, aquaporin-1-positive) (Figure 1D), and increased immune cell signal (Figure 1F and G). DDKT samples showed significantly fewer tubular cells than LDKT biopsies as a proportion of total cell number (*P* = 0.003), with a trend toward reduction in multiple tubular segments, including PT (*P* = 0.04, above significance cutoff corrected for multiple comparisons) (Figure 1H). Deceased donor samples also had a significantly higher proportion of macrophages (*P* = 0.0003) and total immune cells (*P* = 0.001) and, unexpectedly, a higher proportion of endothelial cells (*P* = 0.002) compared with LDKT.

To assess whether these differences reflected absolute changes in cell number, cell populations were compared per unit area of tissue. After correcting for area, deceased donor samples still had significantly fewer tubular cells (*P* < 0.0001), specifically in the PT (*P* < 0.0001) (Figure 1I). Of note, this decrease in tubular cell numbers did not reflect failure to successfully detect nuclei and assign cell identify in the DDKT samples (Figure S2D, SDC, http://links.lww.com/TXD/A333). In the interstitium, DDKT biopsies had significantly more macrophages (*P* = 0.0037) and a trend toward increased total immune cells (*P* = 0.007), whereas the endothelial cell numbers were equivalent in the 2 groups when corrected for area. Without a compensatory rise in interstitial populations, this tubular cell loss drove a significant decrease in total nuclei per mm^2^ in the DDKT group (*P* = 0.0007, data not shown). Overall, these data indicate that high-risk DDKT biopsies showed a significant pretransplant decrease in tubular cell mass and overall cell number with significantly increased macrophage infiltration. Notably, there appeared to be large variance present in the deceased donor population (Figure 1H), raising the possibility of biologically distinct subpopulations.

### Preimplant DGF Biopsies Show Tubular Cell Reduction Compared With Kidneys That Develop IGF

To identify potential biologic drivers of DGF, we subdivided the high-risk DDKT biopsies into those that developed posttransplant IGF or DGF to uncover differences in cell populations that correlated with DGF in this cohort. There were no significant differences in clinical donor characteristics or cold ischemia time between the analyzed IGF and DGF biopsies, though DGF patients trended toward a higher proportion of DCD (4/6 versus 1/5) (Table 2). DGF recipients were more likely to be male (6/6 versus 1/5, *P* = 0.02) and, surprisingly, had significantly lower panel reactive antibody values compared with the IGF patients (0% ± 0% versus 67.2% ± 40.3%, *P* = 0.003) (Table 3). However, it should be noted that samples were analyzed before implantation and exposure to the recipient.

**FIGURE 2.**
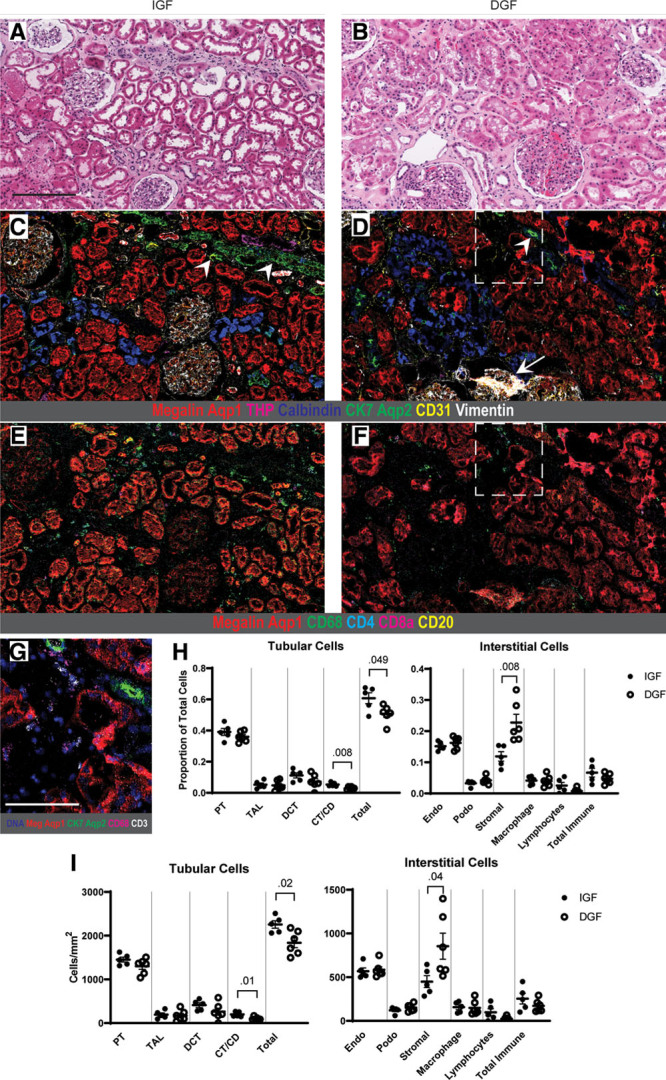
Subgroup analysis of deceased donor kidneys demonstrates reduced tubular cell mass in DGF samples. (A, B) Representative H&E sections from high-risk deceased donor transplants that progressed to IGF (A) and DGF (B). While both had a range of histopathologic injury scoring, there was no clear difference noted between the 2 groups. (C, D) Adjacent tissue sections labeled by IMC, pseudocolored with a selection of antibody channels to highlight tubular and glomerular structures. Note the markedly reduced tubular area and labeling intensity in the DGF sample (D) compared with the IGF section (C) that is not apparent by H&E evaluation alone. This reduction is most notable in the CK7-positive, aquaporin-2-positive connecting tubule/collecting duct, with examples marked by arrowheads. A sclerotic glomerulus is additionally noted in the DGF sample by IMC (arrow). (E, F) The same IMC sections as in (C, D) pseudocolored for immune cell populations. DGF samples did not qualitatively possess more immune cells than IGF biopsies. (G) Inset from (D, F) highlighting CK7-positive, aquaporin-2-positive connecting tubule/collecting duct in the DGF biopsy, with CD68 and CD3 to mark neighboring macrophages and T cells, respectively. (H) Cell proportions of tubular and interstitial cells in IGF and DGF samples. (I) Cells per mm^2^ of analyzed tissue area for tubular and interstitial cells. All *P* values <0.05 shown, and bars show means ± standard errors of the mean. Scale bars: 200 μm (A), 100 μm (G). A gamma value of 1.0 was used for all protein channels in IMC images. Aqp1, aquaporin-1; Aqp2, aquaporin-2; CK7, cytokeratin-7; CT/CD, connecting tubule/collecting duct; DCT, distal convoluted tubule; DNA, DNA intercalator; Endo, endothelium; Meg, megalin; Podo, podocytes; PT, proximal tubule; TAL, thick ascending limb of loop of Henle; THP, Tamm-Horsfall protein.

Morphologically, there was no obvious division in histopathologic scoring for tubular injury or otherwise between the 2 groups (Table 4, Figure 2A and B), consistent with prior studies.^20,35^ IMC, conversely, revealed a comparative reduction in area and labeling intensity of canonical tubular markers as well as a reduction in overall tubular cell number in the DGF samples compared with the IGF group (*P* = 0.02) (Figure 2C versus D, quantification in H-I), most prominently in the connecting tubule/collecting duct (CT/CD; aquaporin-2-positive, cytokeratin-7-positive) (*P* = 0.01) (Figure 2D and G, quantification in H-I). Both groups had comparable immune infiltration with no difference in macrophages or other immune cells (Figure 2E and F, quantification in H-I). DGF samples also trended toward increased stromal cells (*P* = 0.04), which represent a combination of smooth muscle, fibroblasts, pericytes, and other interstitial cell types (Figure 2H and I). Taken together, these data suggest that an absolute reduction in tubular cell mass with a compensatory increase in stromal cells in preimplantation biopsies may differentiate DGF samples from high-risk DDKTs that progress to IGF. While high-risk deceased donor samples showed an overall increase in macrophage infiltration, macrophage or immune cell number was not predictive of subsequent graft function.

### Deceased Donor Biopsies Demonstrate Rare Regenerative Tubular Population by IMC

In our previous study of the healthy human kidney, we identified a small subpopulation of PT cells that coexpressed the intermediate filament vimentin, which typically marks mesenchymal cells such as fibroblasts and pericytes.^36^ Vimentin-positive PT cells have previously been isolated in human and murine models of ischemia and postulated as a phenotypically distinct population of transiently dedifferentiated epithelial cells that may be injured, fibrotic, or regenerative^37-39^ and thus may serve as a precursor to allograft fibrosis.^40^

**FIGURE 3.**
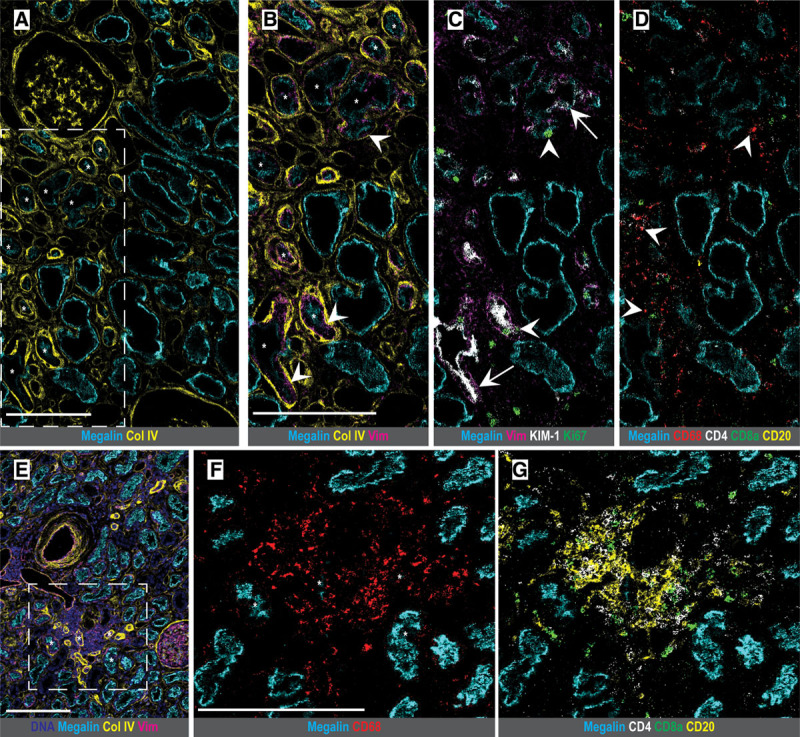
Morphologic inspection of DDKT samples by IMC reveals injured or regenerative tubular population surrounded by immune cells. (A) DGF sample showing proximal tubule with decreased megalin expression surrounded by a thickened basement membrane (collagen IV). Several illustrative tubules are highlighted by stars. (B) Inset of region in (A) showing vimentin coexpression inside the basement membrane and within tubular cells (arrowheads), with the same tubules as in (A) highlighted by stars. (C) Vimentin-positive proximal tubules additionally coexpress the injury marker KIM-1 (arrows) and are enriched for Ki67 nuclear labeling (arrowheads), suggesting proliferation. (D) Tubules are surrounded by a CD68-positive macrophage-rich infiltrate (arrowheads). (E) IGF biopsy with a similar pattern of vimentin-positive proximal tubules with thickened basement membranes (collagen IV) and surrounded by a dense nuclear infiltrate. (F, G) Inset of region in (E) identifying cellular infiltrate as CD68-positive macrophages (F) and lymphocytes (G). Scale bars: 200 μm (A, B, E, F). A gamma value of 1.0 was used for all protein channels. Col IV, collagen IV; DNA, DNA intercalator; KIM-1, kidney injury molecule-1; Vim, vimentin.

Using unsupervised analysis, we identified a discrete vimentin-positive PT cluster in 7/11 DDKT biopsies (3/5 IGF, 4/6 DGF) but in only 1/10 LDKT tissues. These cells comprised 1.66%–10.09% of total PT cells in the biopsies in which they were identified. Qualitative analysis of these cells revealed lower expression of the canonical PT protein megalin as well as thickened basement membranes by collagen IV labeling (Figure 3A and B), both hallmarks of tubular injury.^41,42^ These cells were often simultaneously enriched for the proliferation marker Ki67 (Figure 3C), supporting the hypothesis that they are regenerating cells. We identified these cells in close association with macrophage-rich (Figure 3D) or mixed macrophage and lymphocytic infiltrates (Figure 3E to G). In a subset of tissues, vimentin-positive PTs also coexpressed kidney injury molecule-1 (KIM-1), a marker of PT injury^43^ known to be present in vimentin-positive and proliferative PT cells after ischemia (Figure 3C).^44-46^ These findings suggest that vimentin-positive PT cells may represent a significant target of immune-tubular cross-talk and a source of regenerative capacity in deceased donor organs.

## DISCUSSION

DGF remains a significant clinical challenge with substantial morbidity, yet our lack of mechanistic understanding of underlying cellular injury has limited the development of preventative or curative therapies. IRI and DGF have been associated with multiple mechanisms of tubular cell death.^15,47-49^ However, clinical trials targeting several of these pathways have yielded limited benefit,^50,51^ which underscores the importance of more in-depth tissue analysis of ischemic injury and donor-specific biology. Unfortunately, biopsies are rarely performed in the early posttransplant period, limiting the study of cellular and molecular drivers of DGF. IMC, a high-dimensional immunolabeling technique that our group recently adapted for the human kidney,^28^ substantially increases the throughput and depth of information gained from a single biopsy and can provide an invaluable opportunity to expand our study of transplant injury given the scarcity of available tissue. We have herein presented the application of this methodology to study donor factors contributing to DGF.

In performing unsupervised, quantitative cell clustering analysis, we identified a significant reduction in tubular cells and increased macrophages in high-risk deceased donor kidneys compared with living donors, consistent with the trend toward increased histopathologic tubular injury scores in the DDKT samples. This is in line with mouse models of IRI from our group and others showing that macrophages play a significant role in mediating ischemic tubular injury as well as subsequent reparative or maladaptive responses to that injury.^52-54^ However, there was no difference in immune populations between kidneys that progressed to IGF or DGF, suggesting that donor macrophage number alone does not determine the functional impact of injury. Notably, murine studies have detailed the contrasting roles of proinflammatory (M1) and alternatively activated (M2) macrophages in promoting tubular injury versus repair, and incorporating antibodies to differentiate functionally distinct macrophage subsets may further subdivide the donor macrophage population and better inform our interpretation. Additionally, biopsies were procured before graft implantation, and therefore our analysis can only investigate preimplantation injury without contribution from recipient macrophages or reperfusion.

Of the factors that could be quantitatively assessed by IMC, we found that tubular cell dropout in high-risk deceased donors was most significant in the PT, the segment most prominently damaged by ischemia.^55^ We further uncovered a small population of megalin-low, vimentin-positive PT cells almost exclusively in deceased donor tissue that was surrounded by macrophage-rich infiltrates and coexpressed markers of injury and proliferation, KIM-1 and Ki67. Interestingly, KIM-1 expression has been shown to be protective in murine models of IRI and renal transplant,^56^ and this population may represent surviving PT cells that serve as a source for tubular repair. Murine models suggest that the renal tubule does not have a fixed stem cell population and that regeneration after injury is triggered by stochastic dedifferentiation and division of injured, KIM-1-positive tubular cells.^45,46^ High-dimensional single-cell analysis techniques can play a role in isolating these cells and continue to clarify their viability as a therapeutic target for graft repair.

We identified a trend toward further reduction in tubular cell number in DDKT biopsies that progressed to DGF as compared to other high-risk samples, suggesting that preimplantation tubular cell death may be an important driver of DGF among transplants with similar clinical characteristics. After implantation, tubules that already exhibit dropout may have impaired capability to mount a proliferative response, leading to persistent inflammation and ongoing injury. Because the analyzed DGF cohort was composed of predominantly DCD kidneys, it is a reasonable possibility that ischemia from circulatory death underlies this further decrease in tubular cell number and greater propensity to development of DGF. Additionally, it is noteworthy that this distinction in tubular cell mass between DGF and IGF samples was not apparent using standard histopathologic evaluation for tubular injury, consistent with previous studies of procurement biopsy scoring.^20,35^ This demonstrates that IMC is a more sensitive platform to quantitatively assess renal tubular injury and could provide a key supplement to conventional histopathologic scoring techniques.

With nearly 20% of recovered organs discarded before transplantation^5^ as well as recent data arguing that high-risk kidneys still offer excellent long-term outcomes,^17^ we do not advocate this analysis as justification to discard additional organs. In fact, it should be noted that all grafts included in this study ultimately developed adequate function. However, with turnaround time comparable to a standard immunostaining protocol, IMC labeling of procurement biopsies could be available within 24 hours and thus be used to identify organs that otherwise might not have been known to confer higher risk for enrollment in future trials and to help tailor monitoring and treatment strategies. Additionally, quantitative tissue interrogation by IMC could be implemented to study marginal organs to assess for intact tubular and interstitial cell populations in the hopes of salvaging kidneys for transplant, as this may provide a better correlate with postimplant function than clinical degree of donor kidney injury.^57^

While we identified donor tubular cell differences predisposing to DGF that were not previously detected with conventional histopathologic analysis, our antibody panel did not permit further elucidation of the precise molecular drivers of this tubular loss. Future efforts can incorporate antibody targets for cellular death and stress pathways implicated in DGF and IRI, including apoptosis, autophagy, and necroptosis and regulated necrosis.^15,47-49,58^ Subsequent studies can also more robustly define diverse functional immune subsets and analyze spatial data to better understand the immune interface with the tubular epithelium. Finally, in contrast to the focus on PT injury in IRI, the DGF group unexpectedly had most marked tubular cell loss in the CT/CD. Decreased expression of aquaporin-2 has been shown in some animal models of ischemia,^59,60^ and distal tubular segments may be an underappreciated player in human ischemic injury.^61^ Our work suggests that CT/CD dropout may be a driver in human disease, which beckons further exploration. Additional expansion of our antibody panel to differentiate principal and intercalated cells and to target neutrophil gelatinase-associated lipocalin, a marker of epithelial injury produced in distal tubular segments,^62,63^ can delineate which cell type is predominantly affected and better define the biological significance of this finding.

Limitations of this study include the small patient cohort, which restricted our ability to perform additional multivariate analysis. Technical concerns, including the loss of ~10% of immunolabeled regions as well as the size of our available antibody panel, remain a challenge, and further optimization will be needed to expand the yield of IMC-based analysis in the human kidney. Additionally, due to the relatively short posttransplant follow-up time and absence of postimplantation biopsies, we were unable to characterize the evolution of donor tubular injury in the recipient or the effect of the cellular changes noted on graft outcomes beyond DGF. Prospective study of a larger cohort should be performed for validation and to assess the long-term implications of donor tubular cell loss on graft outcomes.

In summary, we applied quantitative high-dimensional imaging and analysis to preimplantation biopsies from living donors and high-risk deceased donors to investigate ischemic injury preceding DGF. We demonstrated reduced overall tubular and PT cell mass and increased macrophage infiltration in high-risk deceased donor biopsies, and we found a trend toward even greater tubular cell loss, particularly in the CT/CD, in the subgroup that went on to develop DGF as compared to IGF. Additionally, we found a rare population of vimentin-positive, KIM-1-positive, Ki67-positive dedifferentiated PT cells enriched in deceased donor samples as a target for further inquiry of the renal response to donor ischemic injury. This study highlights the potential of IMC to interrogate mechanisms of cell injury and survival and to uncover novel therapeutic targets for both DGF and acute tubular injury.

## ACKNOWLEDGMENTS

We thank Drs Ruth Montgomery and Denis Schapiro for helpful discussion and guidance regarding IMC and data analysis. We acknowledge Shelly Ren and the Yale CyTOF core for technical assistance. We thank Ricarda Tomlin, Dr Sanjay Kulkarni, and the Yale Section of Transplantation Surgery and Immunology for assistance with biopsy acquisition.

## Supplementary Material


