# Supplementary material for: Tubular Cell Dropout in Preimplantation Deceased Donor Biopsies as a Predictor of Delayed Graft Function
Source: Transplant Direct. 2021 Jun 18;7(7):e716. doi: 10.1097/TXD.0000000000001168 (PMC8384397; doi:10.1097/TXD.0000000000001168)
Supplement: Supplementary file 1 [file txd-7-e716-s001.pdf]

Antigen	Target	Species	Clone	Dilution	Metal	Supplier
DNA intercalator	Nucleus	N/A	N/A	1:1000	^{191}Ir	Fluidigm
DNA intercalator	Nucleus	N/A	N/A	1:1000	^{193}Ir	Fluidigm
β-catenin	Tubular epithelium	mouse	D13A1	1:250	^{165}Ho	Fluidigm
Megalin	Proximal tubule	mouse	10D5.1	1:250	^{174}Yb	EMD Millipore
Aquaporin-1	Proximal tubule	rabbit	EPR11588	1:2000	^{173}Yb	Abcam
Tamm-Horsfall protein	Ascending limb of the Loop of Henle	rat	774056	1:1600	^{151}Eu	R&D Systems
Calbindin	Distal convoluted tubule	mouse	401025	1:400	^{142}Nd	Thermo Fisher Scientific
Aquaporin-2	Collecting duct (principal cell)	rabbit	EPR21080	1:300	^{172}Yb	Abcam
Cytokeratin-7	Connecting tubule, collecting duct	mouse	RCK105	1:150	^{164}Dy	Fluidigm
CD31	Endothelium	mouse	JC/70A	1:100	^{149}Sm	Abcam
Nestin	Podocyte	mouse	196908	1:200	^{146}Nd	Fluidigm
α-Smooth muscle actin	Smooth muscle	mouse	1A4	1:1600	^{141}Pr	Fluidigm
Vimentin	Fibroblast, pericyte, mesangium, podocyte	mouse	RV202	1:500	^{150}Nd	Abcam
Collagen IV	Basement membrane, fibrosis	mouse	1042	1:200	^{166}Er	Thermo Fisher Scientific
Renin	Juxtaglomerular cell	rabbit	EPR20693	1:1000	^{171}Yb	Abcam
CD68	Macrophage	mouse	KP1	1:600	^{159}Tb	Fluidigm
CD66b	Granulocyte	mouse	80H3	1:200	^{152}Sm	Fluidigm
CD3	T lymphocyte	polyclonal	polyclonal	1:80	^{170}Er	Fluidigm
CD4	Helper T lymphocyte	rabbit	EPR6855	1:100	^{156}Gd	Fluidigm
CD8a	Cytotoxic T lymphocyte	mouse	C8/144B	1:100	^{162}Dy	Fluidigm
CD20	B lymphocyte	mouse	H1	1:100	^{161}Dy	Fluidigm
Ki67	Proliferation	mouse	B56	1:100	^{168}Er	Fluidigm
KIM-1	Epithelial injury	mouse	219211	1:75	^{160}Gd	R&D Systems

Table S1: IMC antibody panel. KIM-1=kidney injury molecule-1.

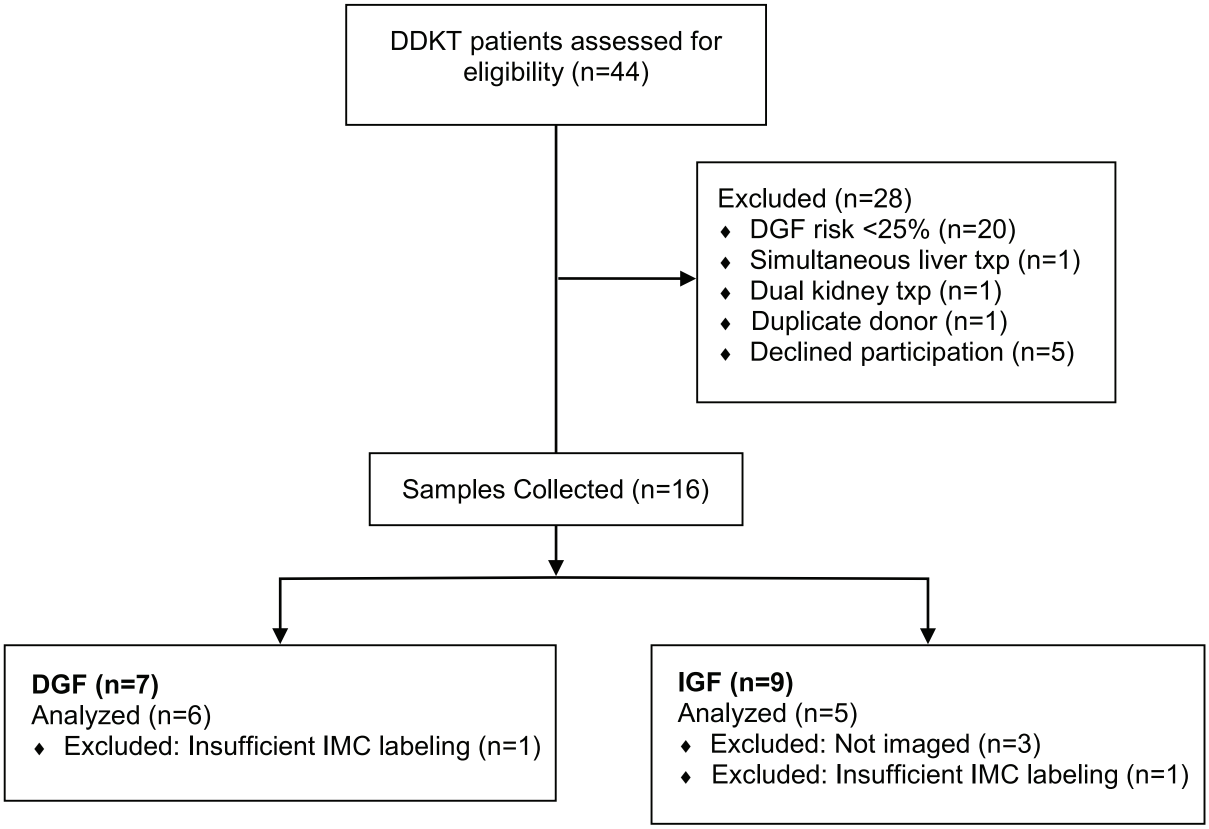

Figure S1: DDKT patient enrollment and sample analysis.

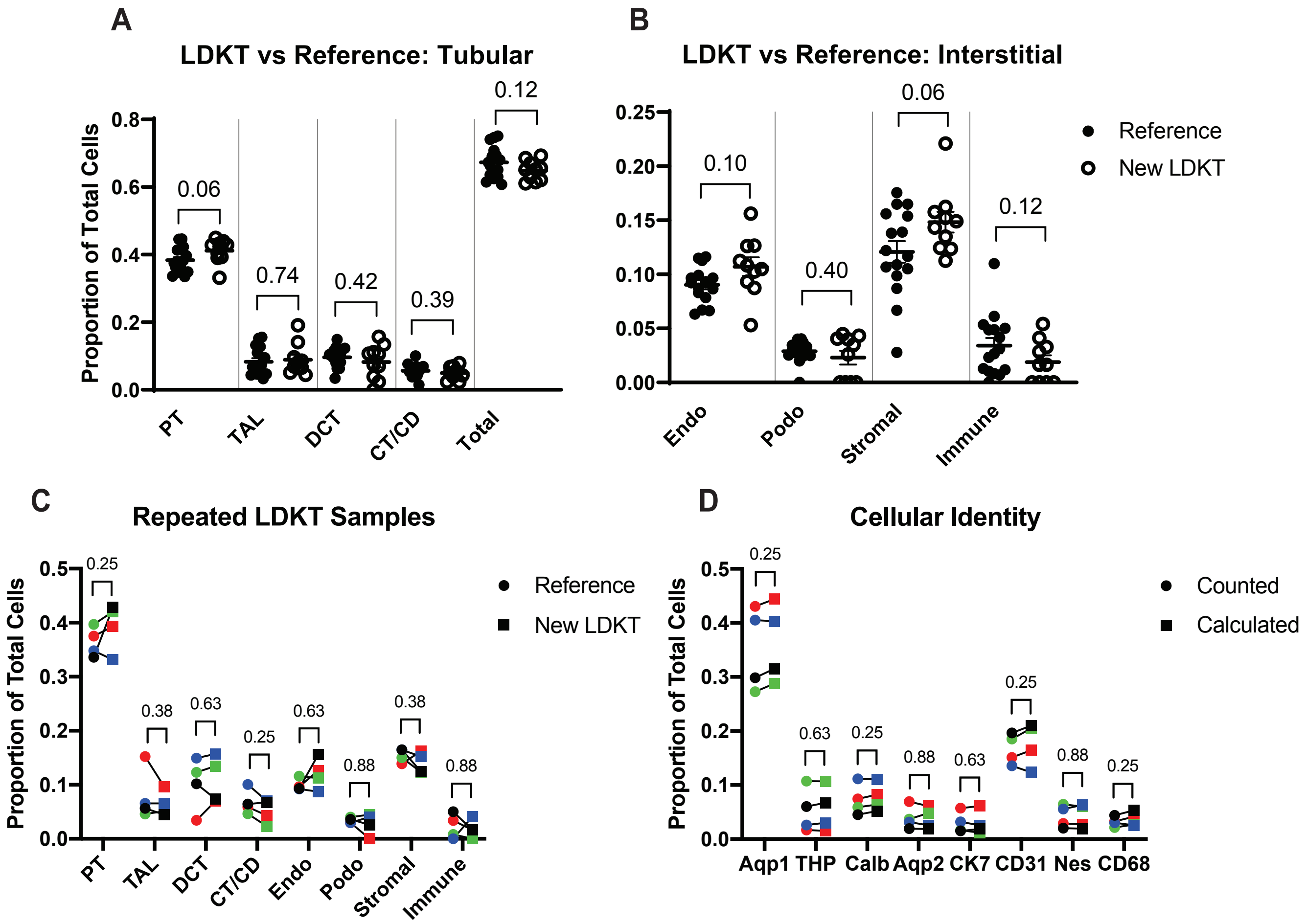

Figure S2: Validation of LDKT cohort and IMC analysis pipeline in injured tissue. (A-B) The current set of 10 LDKT samples was compared to the reference data set in Singh et al.[28] for tubular **(A)** and interstitial **(B)** cell proportions. No significant differences were found in any cell population, with p values shown, confirming the utility of this LDKT set as a control group. **(C)** Four living donor biopsies included in both the reference data set (circles) and the current LDKT panel (squares) were compared for cell proportions, with each biopsy sample shown in a different color. No significant differences were found in any cell population, with p values shown, highlighting the reproducibility of IMC in the human kidney. **(D)** Over 8000 cells from four representative deceased donor regions were manually counted and scored for positivity of eight protein markers defining key tubular, endothelial, glomerular, and immune cell types, followed by comparison to results from the automated pipeline. There were no significant differences between the counted (circles) or calculated (squares) proportions of any cell phenotype, with p values shown. PT=proximal tubule; TAL=thick ascending limb of Loop of Henle; DCT=distal convoluted tubule; CT/CD=connecting tubule/collecting duct; Endo=endothelium; Podo=podocytes; Aqp1=aquaporin-1; THP=Tamm-Horsfall protein; Calb=calbindin; Aqp2=aquaporin-2; CK7=cytokeratin-7; Nes=nestin.
